# Effect of Dispersed Reinforcement on Ultrasonic Pulse Velocity in Stabilized Soil

**DOI:** 10.3390/ma14226951

**Published:** 2021-11-17

**Authors:** Maciej Miturski, Wojciech Sas, Algirdas Radzevičius, Raimondas Šadzevičius, Rytis Skominas, Mateusz Stelmaszczyk, Andrzej Głuchowski

**Affiliations:** 1 Department of Geotechnical Engineering, Institute of Civil Engineering, Warsaw University of Life Sciences—SGGW, 02-787 Warsaw, Poland; 2 SGGW Water Centre, Warsaw University of Life Sciences—SGGW, 02-787 Warsaw, Poland; wojciech_sas@sggw.edu.pl (W.S.); mateusz_stelmaszczyk@sggw.edu.pl (M.S.); 3 Department of Water Engineering, Faculty of Engineering, Vytautas Magnus University Agriculture Academy, 53361 Kaunas, Lithuania; algirdas.radzevicius@vdu.lt (A.R.); raimondas.sadzevicius@vdu.lt (R.Š.); rytis.skominas@vdu.lt (R.S.)

**Keywords:** ultrasonic pulse velocity, soil stabilized, dispersed reinforcement, fibers

## Abstract

One of the fundamental techniques for road subgrades is soil stabilization. Considering the high emission of carbon dioxide during the production of binders, novel techniques to reduce the binder are being studied. Thus, we investigated dispersed reinforcement in stabilized soils. A study was conducted to determine the ultrasonic pulse velocity in nine mixtures of soil, cement, and polypropylene fibers and then correlate the results with other destructive tests. The results show a decrease in wave velocity in mixes with fiber addition by up to 18.5%. The result is dependent on the curing time and whether the samples were stored in a water tank. Immersion in water increases the obtained results by about 6.3%. Based on the analysis, for mixtures with fibers, boundary velocities of waves above which lower values of modulus of elasticity were obtained were determined. Depending on the mix and the module analyzed, the limits range from 2194 m/s to 2498 m/s.

## 1. Introduction

The technique of stabilizing soil for road purposes was already used over 2000 years ago to construct Roman roads [1]. The idea of soil improvement by stabilization is to mix the soft soil with an additive [2], as a result of which its applicability in geotechnical projects will be increased. There are three main approaches in the stabilization process: traditional Stabilizers, by-product stabilizers, or non-traditional stabilizers [3]. Of all the stabilizing products available, cement is the most commonly used to improve the mechanical properties of soil [4,5,6,7]. Cement, which is a binder, bonds soil particles together through a chemical reaction [8,9].

In the present day, there is a continuous increase in the demand for binders such as cement, which is one of the primary materials for stabilizing soil. This has led to a 30-fold increase in cement production since 1950 and an almost four-fold increase since 1990 [10]. The effect of these increases has been to increase the contribution of cement producers to global carbon dioxide (CO_2_) emissions. Today, cement production is the third most significant anthropogenic source of carbon dioxide in the world [11]. By 2050, Europe plans to reduce carbon dioxide emissions into the atmosphere in the cement manufacturing sector [12]. To this purpose, scientific research is being focused on proposing methods for the improvement of soil with simultaneous reduction of cement usage. Current research directions focus mainly on two topics. The first method is based on the reuse of materials from the demolition of concrete structures. Recycled concrete aggregate (RCA) is increasingly used in highway construction, which is the subject of numerous scientific studies [13,14,15,16,17,18]. A second important research direction is the use of soil reinforcement. The first descriptions of the phenomenon were given by Vidal [19] in the mid-20th century. It is assumed that the shear resistance of the obtained composite material will be increased as a result of the applied reinforcement [19,20]. Such soil reinforcement is widely used in embankment design [21,22,23]. Geosynthetics are used for this purpose [24]. Another type of reinforcement is polypropylene fibers, which are dispersed reinforcement. This type of strengthening has been successfully applied in highway engineering [25,26,27]. Current research on the effects of dispersed reinforcement can be divided into two basic types, destructive testing (DT) and non-destructive testing (NDT). Destructive testing involves changing the dimensions of the physical and structural integrity of the specimen. Non-destructive testing, on the other hand, does not affect the structural integrity of the specimen. The material’s mechanical performance can be determined, or defects can be detected without destroying the material [28].

The first type of test includes unconfined compression strength. This is a test commonly used in scientific papers on soils stabilized by cement with the addition of polypropylene (PP) fibers [29,30,31,32,33,34]. This is a destructive test that well-reflects the mechanical properties of the tested material, but has its limitations. A more complex stress state can be obtained from triaxial compression testing. This is another type of destructive testing commonly performed on cement, and polypropylene fiber-reinforced soils [26,35,36,37].

The second type of test carried out is non-destructive tests, which are increasingly used to identify the properties of materials, which is well-illustrated by the dynamic growth of scientific publications in this area [38]. A wide variety of non-destructive techniques and methods are commonly used. Elementary non-destructive testing can include; Microscopy, Radiography, Magnetic Particle, Acoustic emission, and Ultrasonic testing [28,39,40]. These methods are used on composite materials [28], including those used in highway construction, to determine the mechanical properties or to monitor the maturity or curing of cement-stabilized mixtures [41,42]. One of the more popular non-destructive testing techniques is ultrasonic pulse velocity. This method has been used for testing on construction sites since 1960 [40]. UPV testing measures the time it takes for a wave to pass through the material under investigation. There are several types of ultrasound waves, and the most important are compressional waves, also called longitudinal or P-waves. Shear waves are also called transverse or S-waves. Surface waves are also called Rayleigh waves and Lamb waves [40,43]. Additionally, three categories of tests can be separated in the pulse method: pulse-echo, pitch-catch, and through-transmission [43]. UPV testing uses waves with frequencies in the range between 20 kHz and 1 GHz [41]. The time required to travel through the test material depends only on its density and stiffness [44]. Indirectly, the speed of the waves will be affected by the water content of the material [45]. That is why higher wave propagation velocities can be observed in stabilized soils [42,46,47]. Wave velocities are often correlated with unconfined compressive strength [48,49]. The effect of the addition of dispersed reinforcement in stabilized soils on P-Wave and S-wave velocities has already been the subject of research papers [50,51,52]. However, the current state of knowledge requires further research. 

To fully understand the effect of dispersed reinforcement on stabilized soils, it is necessary to know the physical and mechanical properties of the composite material developed. For this purpose, destructive and non-destructive tests are carried out. The full range of tests will allow for determination of not only the compressive and tensile strength of the material, but also the parameters characterizing deformability, including dynamic parameters. In order to determine such values, it is necessary to study the effect of dispersed reinforcement on the velocity of wave propagation in the tested material. This paper investigates the influence of polypropylene fibers on wave velocities. The results are compared with the results for unconfined compressive strength. The presented study shows the effect of curing the material with a cementitious binder on the values obtained. The results were compared with those of other authors.

## 2. Materials and Methods

The chapter is divided into two parts, materials and methods. The material section describes: The clSa soil that is the basis of each mixture is described in Section 2.1.1. The blast furnace cement (BFC) (Górażdże Cement S.A., Chorula, Poland) was used to produce the mixtures. A detailed description is given in Section 2.1.2. Dispersed reinforcement, which is polypropylene fibers, is described in Section 2.1.3. Section 2.1.4 describes the nine mixtures of soil, cement, and fiber that are used. The methods section describes the procedure for sample preparation and care that follows European standards. A detailed description of the procedures is given in Section 2.2.1. The UPV test procedure, measuring device, and instrument calibration are described in Section 2.2.2. The unconfined compression test, measuring device, and calibration are described in detail in Section 2.2.3. Section 2.2.4 describes the analysis between unconfined compressive strength and ultrasonic pulse velocity. Section 2.2.5 describes the analysis between the ultrasonic pulse velocity and elasticity moduli.

### 2.1. Materials

#### 2.1.1. Soil

**Figure 1:**
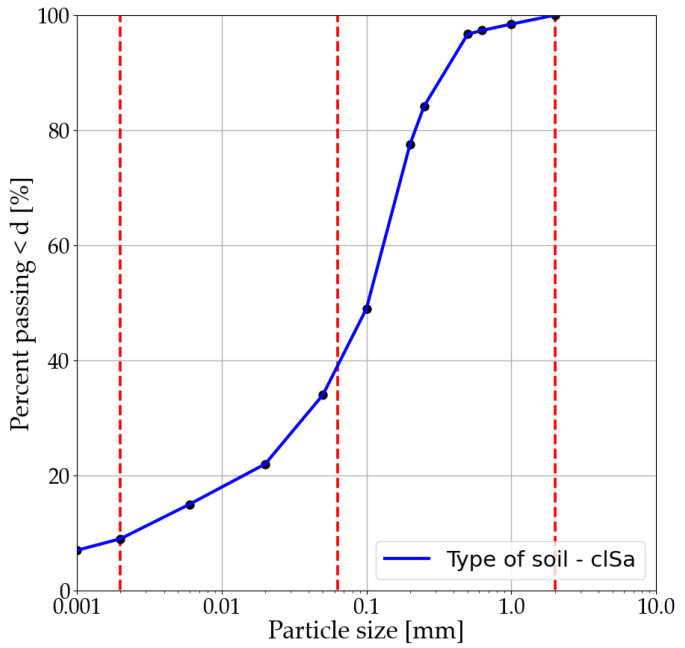
Grain size distribution curve of soil.

The primary component of all the mixtures in this paper is soil taken from a depth of 3.0 m. The soil was studied to determine its physical and mechanical characteristics. An aerometric analysis was conducted using a complete set of sieves, a Casagrande apparatus, and other necessary tests to determine the physical characteristics. The cohesion and angle of internal friction were determined from triaxial compression tests. All tests were conducted following European standards [53]. The main information is presented in Table 1, and the grain size curve is shown in Figure 1. The soil was classified as clayey sand [54].

#### 2.1.2. Binder

Blast furnace cement (BFC) consists of ordinary Portland cement, blast furnace slag and other additives. The blast furnace slag content is up to 65% of the mix [55]. This additive makes it possible to significantly reduce the consumption of Portland cement, which produces a lot of carbon dioxide. An additional advantage is that the cost of such material is reduced because blast furnace slag is a by-product of steel manufacture. Therefore, the use of this type of material as a binder is reasonable.

Blast furnace cement was used to prepare the stabilized soil mixtures. This is a material often used in ground improvement. The material was stored and used before the expiration date stated by the manufacturer. Binder properties are shown in Table 2.

#### 2.1.3. Reinforcement

In this study, polypropylene fibers were used as the reinforcing element of the stabilized soil. All the fibers used have the same length and diameter. The basic information is shown in Table 3.

#### 2.1.4. Mixtures

Nine types of mixtures were used for the study. Each mixture consisted of the soil described in Section 2.1.1, the binder described in Section 2.1.2, and the dispersed reinforcement described in Section 2.1.3. The proportions between the components are shown in Table 4.

### 2.2. Methods

#### 2.2.1. Sample Preparation Procedure and Care Process

All nine mixtures were prepared according to European standards [56]. The binder and reinforcement were measured by weight relative to the weight of the soil. To ensure a homogeneous mix and the best possible fiber distribution, all components were mixed until a homogeneous mix was obtained. The water content was determined individually for each type of mix. All samples were compacted at optimum moisture content and with an energy of 0.59 [J·m^−3^]. The samples were formed in two-part cylindrical molds, the height of the produced samples is 8 cm, and the diameter is 8 cm. The care process followed the European standard [57]. The process itself can be divided into two phases. The first phase involves the care of the samples in a room with constant humidity and a temperature of 22 °C ± 2 °C. In the second phase, the specimens are stored in a water tank until destructive testing. The first phase included 21 days, and the second phase lasted 7 days.

#### 2.2.2. Measurement Devices—Ultrasonic Pulse Velocity (UPV)

Ultrasonic pulse velocity was measured using a Proceq instrument. Pundit lab plus device and 54 kHz transducers were used for the study. Detailed information about the measuring device is presented in Table 5. Before starting the measurements, the device was each time calibrated using the supplied equipment (calibration rod). The device requires calibration each time the cables or transducer frequencies are changed. To properly measure the wave speed, a gel was applied between the transducer and the test sample. The test was performed on each sample for 28 days. During this time, the sample was treated as described in Section 2.2.1. Measurements were performed following European Standards [58,59].

**Figure 2:**
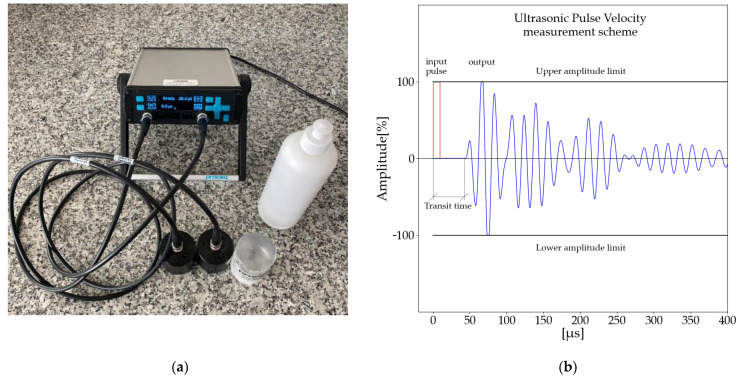
Measuring device: (**a**) Pundit Lab Plus Device, Transducers, Calibration rot; (**b**) UPV test measurement scheme.

**Figure 3:**
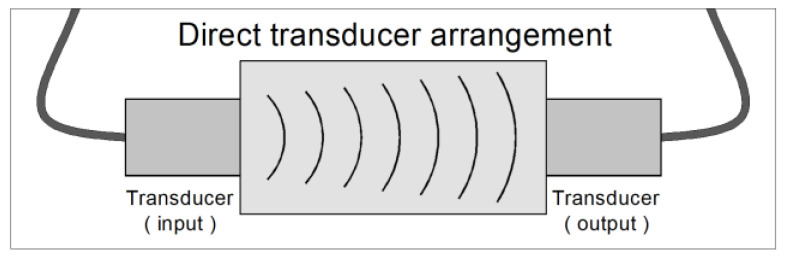
Scheme of the transducer arrangement used in the research.

A scheme of the tests performed is presented in Figure 2. The velocity of the ultrasonic pulse is determined from the relationship:

(1) UPV=LT [ms] 

where *UPV* refers to ultrasonic pulse velocity [m/s], *L* is the distance between two transducers, and *T* denotes transit time. The direct transducer arrangement was used during measurements, Figure 3.

#### 2.2.3. Measurement Devices—Unconfined Compressive Strength (UCS)

The unconfined compression test was performed using an Instron universal testing machine. The machine is equipped with a displacement recorder and load cell. All of the data collected during the test were stored in Bluehill software. The load was applied at a constant rate of 16 kN/min throughout the test. The testing system was calibrated before each test. The main information about the measuring device and the accuracy of the measurements are shown in Table 6.

Unconfined Compressive Strength is determined from the relationship:

(2) $$R_{c}=\frac{F}{A}\;\left[MPa\right]\;$$

where $R_{c}$ refers to the unconfined compressive strength [MPa], *F* is the ultimate applied force, and *A* is the specimen area.

#### 2.2.4. Non-Linear Relationship between Ultrasonic Pulse Velocity and Compressive Strength

The ultrasonic pulse velocity and unconfined compressive strength results obtained were used for analysis. Based on the obtained results, a non-linear analysis was performed. The result of the analysis is the determination of parameters to the exponential trend line, and the line shows the relationship of ultrasonic pulse velocity and unconfined compressive strength. The relationship is defined by the equation:

(3) $$UCS_{calc}=\alpha \cdot e^{\beta \cdot UPV}\;\left[\mathrm{MPa}\right]\;$$

where $UCS_{calc}$ refers to the predicted compressive strength value [MPa], α is the first constant parameter to the exponential function, β is the second constant parameter to the exponential function, *UPV* is the ultrasonic pulse velocity, and *e* is the Euler’s number.

#### 2.2.5. Relationship between Ultrasonic Pulse Velocity and Elasticity Moduli

In the following section, the non-linear relationship presented in the previous Section is analyzed with the linear relationship of the elastic modulus and unconfined compressive strength. The linear relationship presented in the previous work was used in the analysis [34]. Two types of elasticity modules were selected for analysis: $E_{s}$—secant modulus, and $E_{50}$—secant moduli determined for half ultimate unconfined compressive strength. 

**Figure 4:**
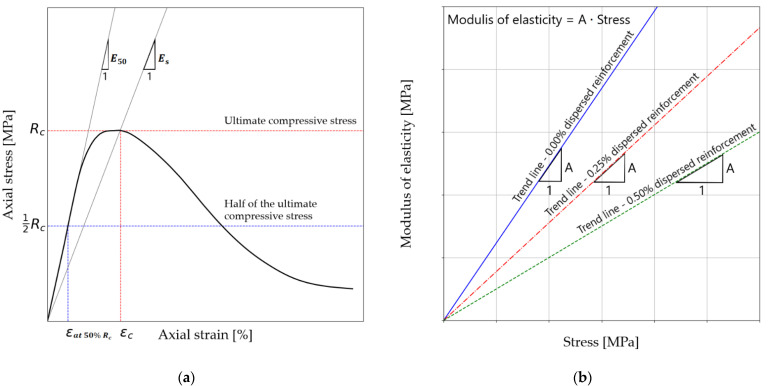
(**a**) Elasticity modules under consideration; (**b**) linear relationship scheme showing the effect of dispersed reinforcement.

The analysis is intended to allow the determination of modulus values from ultrasonic pulse velocity measurements. Figure 4 shows the considered moduli and the linear correlation scheme between the considered modulus values and compressive strength. For analysis purposes, the linear relationship shown in Equation (4) has been transformed.

(4) $$E_{\left(s,\;50\right)}=A\cdot R_{c}\;\left[MPa\right]\;$$

where $R_{c}$ is the unconfined compression strength, and *A* is the slope of the trend line.

The measured value of the unconfined compressive strength is replaced by the predicted value of the unconfined compressive strength expressed by Equation (3). After substitution, the equation was obtained:

(5) $$E_{\left(s,\;50\right)UPV}=A\cdot \alpha \cdot e^{\beta \cdot UPV}\;\left[MPa\right]\;$$

which can be expressed as:

(6) $$E_{\left(s,\;50\right)UPV}=A\cdot UCS_{calc}\;\left[MPa\right]\;$$

where $E_{\left(s,\;50\right)UPV}$ is the predicted value of moduli, *A* is a parameter characterizing the slope of the linear relationship between modulus of elasticity and stress, $UCS_{calc}$ is the predicted compressive strength.

## 3. Results

The research and results presented were conducted according to the procedures described. The chapter was divided into three subsection. The first Section presents the results obtained from the ultrasonic pulse velocity measurements. Wave velocity results for the 7th and 28th day of curing are summarized in the form of bar charts, and then example waves for each mixture are presented. At the last part of the analysis the change of ultrasonic pulse velocity during curing of stabilized soil is presented. The second Section presents the results of the non-linear analysis, presents the relationships in graphical form, and tables the results. The third Section presents the analysis of the obtained results with the results from the previous study, on the basis of which the relationships between the ultrasonic pulse velocity and elasticity moduli were determined.

### 3.1. Effect of Dispersive Reinforcement on Ultrasonic Pulse Velocity

The velocity of elastic waves in solids depends on the interaction forces between atoms and the mass of atoms transmitting the wave motion. The use of dispersed reinforcement in the form of polypropylene fibers changes the structure of such a composite, which affects the speed of wave propagation in the medium. Composites in terms of structure and properties are very different. Therefore, the speeds will depend on the type of matrix used, the type of reinforcement, or the day of curing. The test itself may be subject to measurement error due to uncontrolled fiber distribution. There may be places where dispersed reinforcement has accumulated, as well as places where there is less of it.

**Figure 5:**
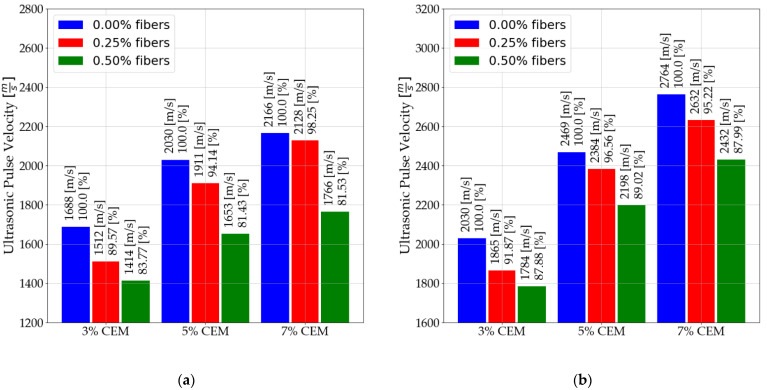
Ultrasonic pulse velocity: (**a**) after 7 days of mixture curing; (**b**) after 28 days of mixture curing.

Tests were conducted for nine mixtures. For each mixture, at least 10 tests were performed. The results presented show the averaged values. Measurements of ultrasonic pulse velocity were carried out for 28 days, and the first measurements were made after 24 h from compaction. The first 21 measurements were made during the first phase of the curing period. The next seven measurements were taken during the second curing phase. A summary for days 7 and 28 is shown in Figure 5.

**Figure 6:**
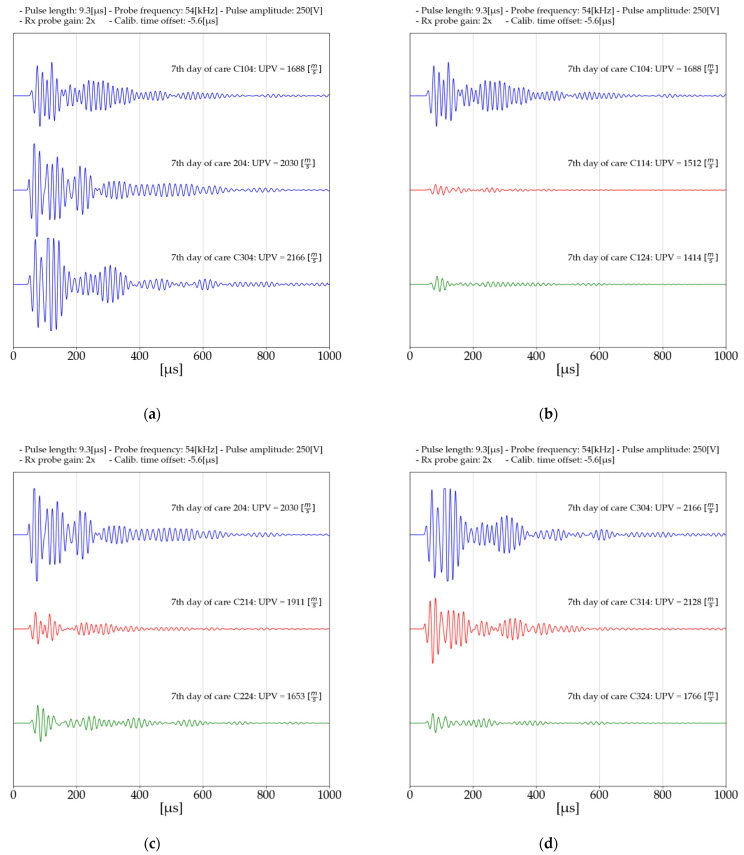
Received waves during Ultrasonic pulse velocity test—day 7 of curing period: (**a**) Mixtures without polypropylene fibers; (**b**) mixtures with 3% binder; (**c**) mixtures with 5% binder; (**d**) mixtures with 7% binder.

Figure 6 shows the received waves during the seventh day of curing. The details of the sent pulse are shown in the graph. The presented results were obtained with the same configurations. Thus, the effect of distributed reinforcement on the received waves can be accurately observed.

**Figure 7:**
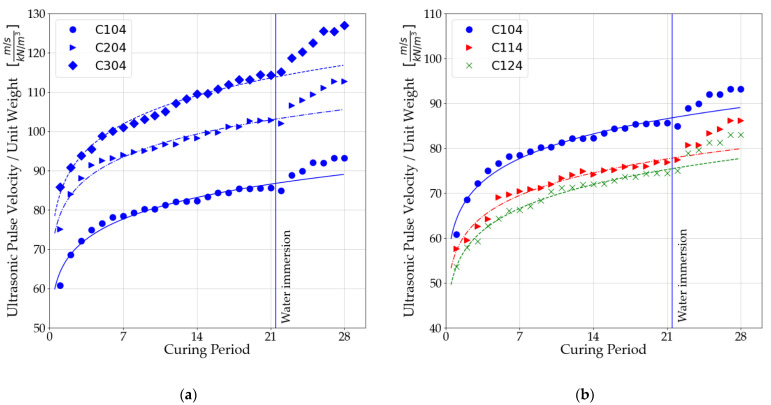

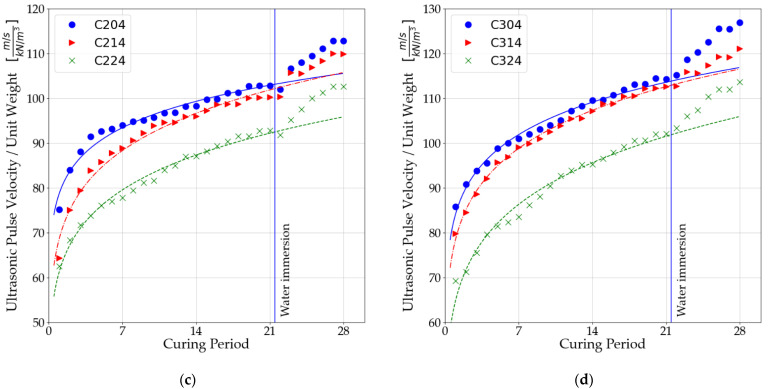
Influence of curing period on wave velocity, normalized by the unit weight: (**a**) Mixtures without polypropylene fibers; (**b**) mixtures with 3% binder; (**c**) mixtures with 5% binder; (**d**) mixtures with 7% binder.

By using a material that is more ductile than the soil-cement matrix, it creates a softer region along the wave travel path, constantly reducing the energy of the wave [51]. A similar effect has been observed by other authors during bender element testing [52], as well as with ultrasonic pulse velocity (UPV) testing [51]. The phenomenon is indirectly related to the change in density of the soil matrix of the binder and fibers. This density decreases as a result of the applied reinforcement. This is caused by the replacement of soil and binders in the mixture by fibers that have a lower density, which reduces the density of the whole mixture. It has been observed that there may be a specific fiber content that will increase the density but not decrease it. To make the results density independent, the ultrasonic pulse velocities normalized by the unit weight of the mixture, which is shown in Figure 7.

It should be noted that by using fibers we reduce the contact area between the particles, which are replaced by connections between the particles and the dispersed reinforcement. An important factor in the cement and reinforcement soil matrix is the strength of the connections between the particles or particles and fibers. It rises as a result of using more binder. As a result of the growth of the connection strength, less benefit is noted from the use of fiber dispersed reinforcement. If the connections between the particles are strong enough, a weakness of the matrix may occur due to the increasing contact area between fibers and particles. The weakening will be characterized by reduced strength as well as modulus values. Such a phenomenon has been observed in previous studies [60]. Therefore, there must be some limit to the applicability of the reinforcement dispersed in the soil and cement matrix. Above this limit, weakening of the material will be noted. Below this limit, a strengthening of the composite material is obtained. 

Based on the results presented, the following relationships can be observed:

- As the amount of binder in stabilized soils increases, the ultrasonic pulse velocity increases. This is due to the effect of the binder on the bonds produced between the particles of the manufactured matrix.
- A significant effect of fibers on the soil–cement matrix is a reduction in wave velocity. This is directly related to the increased number of interfaces in the wave propagation path [51]. In the obtained results, decreases in wave speed are noticeable, regardless of the amount of binder used. It is important to note that stronger connections are formed between the particles as the mixture is cured, which translates directly into reduced wave velocity reductions. Ultrasonic pulse velocity for mixtures with 5% binder content. It decreased to 81.43% after 7 days of curing and only to 89.02% after 28 days of curing.
- The use of dispersed reinforcement in stabilized soils also affects the received waves. A decrease in amplitude can be observed in samples with polypropylene fibers.
- An increase in ultrasonic wave velocity was observed when the samples were immersed in a water tank. Where a slight decrease in wave velocity was observed. From Figure 7, you can interpret the differences due to the immersion of the samples in water. The differences are presented in Table 7.

### 3.2. Results of Analysis of the Relationship between UPV and UCS

Non-linear analysis was performed to determine the relationship between ultrasonic pulse velocity (UPV) and unconfined compressive strength (UCS). For this purpose, parameters were determined for Equation (3), the non-linear regression line. Parameters were determined for:

- Soil-binder matrix, without polypropylene fibers,
- Soil-binder-fiber matrix, polypropylene fibers in the amount of 0.25%,
- Soil-binder-fiber matrix, polypropylene fibers in an amount of 0.50%.

The analysis was conducted after the 28th day of mixture curing. Mean wave velocities and compressive strengths were determined from the tests. The determined parameters α and β to Equation (3) are summarized in tabular form, Table 8. The results are presented depending on the amount of dispersed reinforcement used.

**Figure 8:**
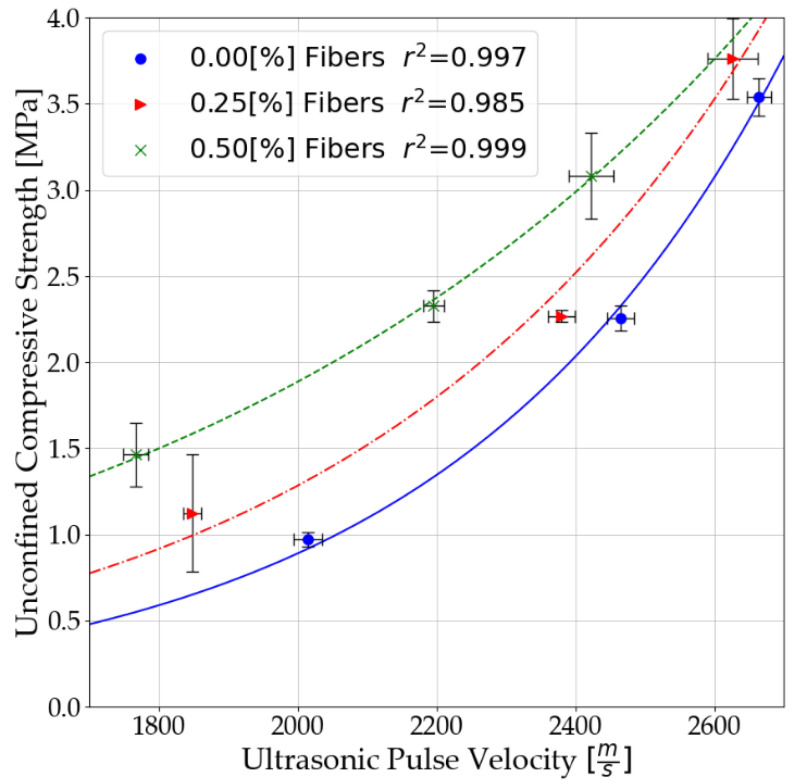
Relationship of ultrasonic pulse velocity and unconfined compressive strength after 28 days of curing of mixtures.

From the parameters presented, it is possible to determine the non-linear relationship between unconfined compressive strength and ultrasonic pulse velocity. The relationships for cement-stabilized soils without and with dispersed reinforcement and the standard deviation of the obtained results are presented in Figure 8.

Based on the analysis, the strengthening of the soil–cement matrix is evident. At higher wave speeds, the fiber improvement is less, which is directly related to the higher amount of binder used. This effect is due to the strengthening of particle and fiber connection forces. It is important to note that it is not only the amount of binder used that has an impact. The type of soil, the ratio of water to the binder, and the care conditions are also important factors. However, it can be assumed that with increasing binder content, the influence of polypropylene fibers will be less and less visible. 

### 3.3. Results of Analysis of the Relationship between UPV and Elasticity Moduli

To analyze the effect of ultrasonic pulse velocity (UPV) on the values of $E_{s}$—secant modulus, and $E_{50}$—secant moduli determined for half ultimate unconfined compressive strength. Equations (5) and (6) were used, which are described in Section 2.2.5. The analysis was carried out separately for mixtures without polypropylene fibers and for contents equal to 0.25% and 0.50%. For this purpose, the parameters determined in Section 3.2 were used. In this way, the following equations were obtained:

(7) $$E_{\left(s,\;50\right)UPV}{}_{0.00\%\;Fiber}=A\cdot 0.01429578\cdot e^{0.00206532\cdot UPV}\;\left[\mathrm{MPa}\right]\;$$

(8) $$E_{\left(s,\;50\right)UPV}{}_{0.25\%\;Fiber}=A\cdot 0.04414984\cdot e^{0.0016846\cdot UPV}\;\;\;\left[\mathrm{MPa}\right]$$

(9) $$E_{\left(s,\;50\right)UPV}{}_{0.50\%\;Fiber}=A\cdot 0.18927741\cdot e^{0.00114934\cdot UPV}\;\left[\mathrm{MPa}\right]$$

where parameter *A* is described in Section 2.2.5. Its values are based on research [34], and shown in Table 9.

**Figure 9:**
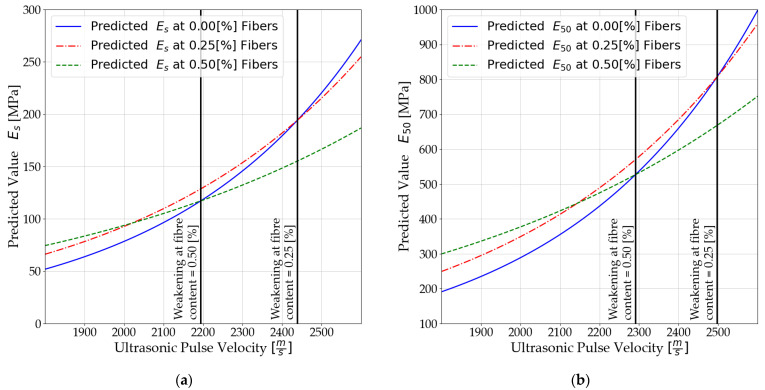
Non-linear relationship between ultrasonic pulse velocity and: (**a**) the $E_{\;s}$; (**b**) the $E_{50}$.

Three curves were obtained based on the analysis. The limiting velocities of ultrasonic pulses at which the analyzed modules reach lower values in relation to mixtures consisting of soil and cement were analyzed and determined. The results are presented in Figure 9.

Figure 9 clearly shows the limiting velocities of ultrasonic pulses beyond which the deformation parameters of stabilized soils begin to increase more slowly, and at the same time, reaching lower values than mixtures without the addition of dispersed reinforcement. For mixtures with a fiber content equal to 0.25%, the limits are as follows:

- 2439 [m/s] for $E_{s}$—secant modulus;
- 2498 [m/s] for $E_{50}$—secant moduli determined for half ultimate unconfined compressive strength.

For mixtures with fiber content equal to 0.50%, the limits are as follows:

- 2194 [m/s] for $E_{s}$—secant modulus;

- 2291 [m/s] for $E_{50}$—secant moduli determined for half ultimate unconfined compressive strength.

In addition, it should be noted that there is a value of ultrasonic pulse velocity at which mixtures with 0.50% of dispersed reinforcement reach lower values of modulus of elasticity than those with 0.25%.

## 4. Discussion and Conclusions

Due to the complex structure of the composite material, measurement technique, and data interpretation, the results obtained may be different from those of other authors [51,52]. However, the data presented in the paper confirms the general trend of the influence of dispersed reinforcement on the ultrasonic pulse velocities obtained. The relationships between unconfined compressive strength (UCS), ultrasonic pulse velocity (UPV), and modulus of elasticity: $E_{s}$—secant modulus, and $E_{50}$—secant moduli determined for half of the ultimate unconfined compressive strength were presented in this paper. Polypropylene fibers in soils stabilized with blast furnace cement cause a number of changes. Fundamental changes occur in the physical and mechanical properties. With regard to physical properties, it is worth noting that the use of dispersed reinforcement reduces the density of the mixture and increases its optimum moisture content. Such phenomena for the studied mixtures were described in the paper [34]. The influence of dispersed reinforcement on mechanical properties, such as unconfined compressive strength and modulus of elasticity dependence on failure stresses, was also described in the paper [34]. The results presented focus on the effect of polypropylene fibers on ultrasonic pulse velocities and its correlation with other properties of soil, cement, and fiber mixtures.

The study found that the ultrasonic pulse velocity decreases in a material with dispersed reinforcement, which is caused by the different stiffnesses of fibers and matrix. The wave must pass through the contact zone many times. As a result of these passes, a decrease in velocity and amplitude can be observed. Note the distribution of reinforcement, which is uncontrolled. This can cause local clusters of fibers or areas where the fiber content is reduced. In addition, their distribution itself is random. In the general case, such material can be treated as isotropic. Comparing the results obtained with the studies carried out by other authors, attention should be paid to the different measurement techniques. The presented wave velocity normalized by unit weight obtains values more than three times higher than the results presented by other authors [51]. This may be due to the use of different base materials, soil, and the ratio of water to cement. However, the overall effect of reinforcement shows a similar trend. The paper points out that there may be a certain amount of fiber at which higher wave velocities can be obtained. 

The results presented in this paper indicate that there may be an optimum amount of reinforcement above which the opposite effect can be obtained. As the cement content increases, it becomes the leading factor affecting the compressive strength. Dispersed reinforcement applied in a lower strength matrix results in increased elastic modulus values. This effect begins to decrease as the strength increases. Beyond the limit of applicability, the dispersed reinforcement begins to have a negative impact on the reinforced material. There is a reduction in the elastic modulus. This effect intensifies with increasing polypropylene fiber content. In this paper, the limits of applicability were determined using ultrasonic pulse velocity tests. The presented approach will allow for determination of the extent of reinforcement of the manufactured material without destructive testing. Non-destructive testing can be used to inspect the produced ground improvement.

Based on the results obtained from tests conducted on nine types of mixes, consisting of soil, blast furnace cement (BFC), and polypropylene fibers, the authors note that:

- The use of dispersed reinforcement in the soil–cement matrix leads to a decrease in the velocity of the passing waves. In addition, lower values of the amplitudes of the received waves were noted. This suggests that the application of dispersed reinforcement also has an effect on wave attenuation.
- There is a limit to the reasonable use of dispersed reinforcement. Below this limit, increased compressive strength and higher elastic moduli of the matrix can be achieved. Beyond the limit of applicability of the reinforcement, the effect of increased compressive strength will be less noticeable. In addition, a reduction in elastic modulus values may occur. This aspect can be very important from a practical point of view. Knowing the limiting wave velocities in the tested material will allow a simple estimation of whether and to what extent we have increased not only the unconfined compressive strength but also the values of moduli of elasticity. Using the optimum amount of fiber will allow a lower amount of cement to be used.
- The type of curing used affects the results obtained. This is well illustrated by increasing the ultrasonic pulse velocity after the 21st day of mix curing. Knowing the effect of the type of curing on the soil stabilized with dispersed reinforcement may lead to misdiagnosis of the tested material. Currently, there are several types of applied treatment in stabilized soils. They differ in the time period spent in the water tank and the length itself. Further research would need to investigate the effect of different times of immersion of samples in water on the wave velocities obtained. This approach would allow correlating the ultrasonic pulse velocity with the moisture content of the mixtures. This may enable the application of the presented measurement method in the field.

The effect of dispersed reinforcement on stabilized soils requires further research. The following are some of the issues that need to be investigated:

- Effect of reinforcement type and length on ultrasonic pulse results. There are many types of dispersed reinforcement. To understand the impact of other materials, such as reinforcement made with recycled materials, this material reuse can significantly impact environmental protection.
- Influence of the basic materials that make up the matrix. The impact of different binders, soils, or the influence of moisture alone must be examined.
- Conduct field tests to verify the experiments performed. The key question is whether this method of improving mechanical properties can be used under field conditions. If so, will the improvement effect be similar to laboratory effects.

In addition, many other factors need to be analyzed, such as the effect of cyclic loading on the material’s behavior over the life of the designed road, and the effect of distributed reinforcement on dynamic parameters. Knowledge of such parameters can be useful in developing guidelines for designers.

## Figures and Tables

**Table 1:** Properties of the soil.

Physical Properties of Soil	Value	Units
C_C_	4.60	[‒]
C_U_	52	[‒]
PI	10.41	[‒]
LI	0.31	[‒]
W_L_	18	[%]
W_p_	7.59	[%]
W	10.8	[%]
pH	8.95	[‒]
MDD	2.08	[g·cm^−3^]
OMC	8.64	[%]
Cohesion	7	[kPa]
Internal friction angle	34.5	[°]

**Table 2:** Properties of Blast furnace cement.

Properties of Binder	Value	Units
Required compressive strength after 7 days	≥16.0	[MPa]
Required compressive strength after 28 days	≥32.5	[MPa]
	≤52.5	[MPa]
Hydration heat	≤270	[J/g]

**Table 3:** Properties of polypropylene fibers.

Properties of Fibers	Value	Units
Single fiber length	12	[mm]
Diameter of a single fiber	32	[µm]
Tensile strength	300 ÷ 400	[MPa]

**Table 4:** Overview of the mixtures.

Mixture Name	Binder [%]	Fibers [‰]	OMC [%]	MDD [g·cm^−3^]
C104	3	0	9.9	1.99
C114	3	2.5	10.2	1.96
C124	3	5	10.9	1.96
C204	5	0	10.1	1.99
C214	5	2.5	10.35	1.97
C224	5	5	11.1	1.96
C304	7	0	11.1	1.96
C314	7	2.5	11.7	1.95
C324	7	5	12.1	1.93

**Table 5:** Measuring device parameters.

Parameter	Value	Units
Range	0.1–9999	[µs]
Resolution	0.1	[µs]
Frequency	24, 37, 54, 82, 150, 200, 220, 250, 500	[kHz]
frequency used	54	[kHz]
Pulse width	9.3	[µs]
Excitation voltage	125, 250, 350, 500	[v]
Used Excitation voltage	250	[v]
Calib. Time offset	−5.6	[µs]

**Table 6:** The main information about the instrument and measurement.

Details	Value	Units
Model	5982	[‒]
Force Measurement Accuracy	±0.5	[%]
Displacement Measurement Accuracy	±0.01	[mm]

**Table 7:** Effect of water on ultrasonic pulse velocities, normalized by the unit weight.

	Ultrasonic Pulse Velocity/Unit Weight [mskNm3]		
Mixture Name	Without Immersion in Water	With Immersion in Water	[%]
C104	89.075	93.189	104.62
C114	79.908	86.194	107.87
C124	77.716	83.002	106.80
C204	105.572	112.782	106.83
C214	105.757	109.939	103.95
C224	95.866	102.601	107.03
C304	116.872	126.954	108.63
C314	116.533	121.098	103.92
C324	105.942	113.641	107.27

**Table 8:** Parameters for the exponential function.

Mixture Name	Binder [%]	Fibers [‰]	α [‒]	β [‒]
C104	3	0		
C204	5	0	0.01429578	0.00206532
C304	7	0		
C114	3	2.5		
C214	5	2.5	0.04414984	0.0016846
C314	7	2.5		
C124	3	5.0		
C224	5	5.0	0.18927741	0.00114934
C324	7	5.0		

**Table 9:** Parameter ***A*** for determining the prediction of modulus values for stabilized soils.

Fibers Content of the Stabilized Soil [‰]	E (Type) [MPa]	*A* [‒]	***r***^2^ [‒]
0.0	$E_{s}$	88.257	0.880
0.0	$E_{50}$	324.785	0.875
2.5	$E_{s}$	72.324	0.807
2.5	$E_{50}$	272.177	0.809
5.0	$E_{s}$	49.734	0.883
5.0	$E_{50}$	200.018	0.710

## Data Availability

The data presented in this paper are available upon request from the authors. These data are not publicly available due to ongoing follow-up work.
